# Various applications of TALEN- and CRISPR/Cas9-mediated homologous recombination to modify the *Drosophila* genome

**DOI:** 10.1242/bio.20147682

**Published:** 2014-03-21

**Authors:** Zhongsheng Yu, Hanqing Chen, Jiyong Liu, Hongtao Zhang, Yan Yan, Nannan Zhu, Yawen Guo, Bo Yang, Yan Chang, Fei Dai, Xuehong Liang, Yixu Chen, Yan Shen, Wu-Min Deng, Jianming Chen, Bo Zhang, Changqing Li, Renjie Jiao

**Affiliations:** 1 State Key Laboratory of Brain and Cognitive Science, Institute of Biophysics, the Chinese Academy of Sciences, Beijing 100101, China; 2 University of Chinese Academy of Sciences, Beijing 100080, China; 3 Key Laboratory of Cell Proliferation and Differentiation of the Ministry of Education, College of Life Sciences, Peking University, Beijing 100871, China; 4 Department of Biological Science, Florida State University, Tallahassee, FL 32304-4295, USA; 5 The Key Laboratory of Marine Genetic Resources, The Third Institute of Oceanography, State Oceanic Administration, Xiamen 361005, China

**Keywords:** *Drosophila*, TALEN, CRISPR/Cas9, Homologous recombination, Targeted genomic modification

## Abstract

Modifying the genomes of many organisms is becoming as easy as manipulating DNA in test tubes, which is made possible by two recently developed techniques based on either the customizable DNA binding protein, TALEN, or the CRISPR/Cas9 system. Here, we describe a series of efficient applications derived from these two technologies, in combination with various homologous donor DNA plasmids, to manipulate the *Drosophila* genome: (1) to precisely generate genomic deletions; (2) to make genomic replacement of a DNA fragment at single nucleotide resolution; and (3) to generate precise insertions to tag target proteins for tracing their endogenous expressions. For more convenient genomic manipulations, we established an easy-to-screen platform by knocking in a *white* marker through homologous recombination. Further, we provided a strategy to remove the unwanted duplications generated during the “ends-in” recombination process. Our results also indicate that TALEN and CRISPR/Cas9 had comparable efficiency in mediating genomic modifications through HDR (homology-directed repair); either TALEN or the CRISPR/Cas9 system could efficiently mediate *in vivo* replacement of DNA fragments of up to 5 kb in *Drosophila*, providing an ideal genetic tool for functional annotations of the *Drosophila* genome.

## INTRODUCTION

In the past two years, geneticists and molecular biologists have realized the emergence of two fascinating genetic manipulation techniques, which have been shown to be applicable in essentially all animals and plants. The earlier method is based on an arbitrary transcription activator-like effector nuclease (TALEN) system (Cermak et al., 2011; Huang et al., 2011; Liu et al., 2012; Cheng et al., 2013; Friedland et al., 2013; Li et al., 2013; Sung et al., 2013; Wang et al., 2013; Yu et al., 2013a). A pair of customizable TALENs needs to be designed for a particular genomic locus that is designated for modifications (Miller et al., 2011; Zhang et al., 2011). Very recently, another method has been quickly adapted for genomic modifications in many organisms (Friedland et al., 2013; Li et al., 2013; Wang et al., 2013; Yu et al., 2013a), which is based on the Cas9 nuclease and a single guide RNA (gRNA) from the type II bacterial clusters of regularly interspaced short palindromic repeats (CRISPR) system (Jinek et al., 2012). Both of these methods rely on a nuclease, FokI or Cas9, to cut the genomic DNAs, which triggers the cellular repair pathways of double strand DNA breaks (DSBs) via either non-homologous end joining (NHEJ) or homology-directed repair (HDR). The targeted DNA binding specificity is determined by TALE repeats through protein–DNA contact in TALEN-mediated genetic modifications, whereas in the case of a CRISPR/Cas9 system, the specificity is determined by the gRNA binding through RNA–DNA contact (Miller et al., 2011; Zhang et al., 2011; Jinek et al., 2012).

In *Drosophila*, our previous studies and work from other fly labs have successfully established both TALEN and CRISPR/Cas9 techniques in manipulating the fly genome (Liu et al., 2012; Bassett et al., 2013; Gratz et al., 2013; Yu et al., 2013a). Most of these studies have focused on generating indels (insertions and/or deletions) at specific loci, while a few labs have very recently reported HDR mediated genome modifications by either the TALEN (Katsuyama et al., 2013) or CRISPR/Cas9 system (Baena-Lopez et al., 2013; Gratz et al., 2013). The size of indels is not controllable with standard TALEN or CRISPR/Cas9 methods through the NHEJ pathway, and can vary from one nucleotide to over hundreds of base pairs (Huang et al., 2011; Yu et al., 2013a). Although sufficient for generating mutations of a gene, unpredictable sizes of indels are not appropriate for making precise deletions and insertions or making replacement with designed mutations across the genome; modifications that are more useful for *in vivo* functional studies. These goals can be achieved only through the HDR pathway by addition of a homologous donor sequence while injecting either TALEN or CRISPR/Cas9 RNAs.

In this paper, we report a series of efficient applications derived from HDR-mediated genomic modifications by TALEN and CRISPR/Cas9 in manipulating the *Drosophila* genome to precisely: (1) generate deletions of the micro RNAs, specifically, *miR-281*; (2) make genomic replacement of endogenous sequences of *CG4221*, *chameau* and *CG5961* genes with exogenous *loxP* sites or restriction enzyme cutting sites of SmaI and HindIII, respectively; and (3) insert coding sequences of GFP and Myc to tag the Chameau and CG4221 proteins for tracing their endogenous expressions. We also established an easy-to-screen platform for more convenient genome-wide genetic manipulations and provided a strategy to remove, if necessary, unwanted duplications generated during the “ends-in” recombination process. Comparing with what has been reported very recently in the literature (Gratz et al., 2013), we achieved a much higher efficiency of HDR by using *Lig4* mutant flies as recipients for injection; we directly inject DNA plasmids instead of single-strand oligonucleotides, thus our approach is more practical for donor preparation, especially when longer homologous sequences are needed.

## RESULTS

### TALEN-mediated precise mutagenesis via the HDR pathway

The first application we sought to explore for TALEN and CRISPR/Cas9 induced HDR in *Drosophila* was to generate precise mutagenesis in the genome. To achieve this purpose, we took advantage of *Ligase4* mutant (*Lig4^169^*) embryos for microinjection, because loss of function of the *Ligase4* gene blocks NHEJ mediated double strand break (DSB) repair and thus promotes the HDR pathway (Beumer et al., 2008; Bozas et al., 2009; Beumer et al., 2013). HDR induced precise mutagenesis is particularly useful for generating null mutations of microRNAs and other non-coding RNAs, and for those genes with multiple splicing isoforms. Here, for the TALEN-mediated HDR mutagenesis, we selected two *Drosophila* genomic loci, *miR-281* and *chameau*. *miR-281* consists of two adjacent miRNAs, *pre-miR-281-1* and *pre-miR-281-2* (Xiong et al., 2009), the functions of which remain unknown. A mutant allele for the long isoform of *chameau* has been reported (Grienenberger et al., 2002), in which the short isoform seems to be not affected. We set out to generate a mutant allele that uncovers both the long and short isoforms of *chameau* in order to get a null mutant of the *chameau* gene.

**Fig. 1.**
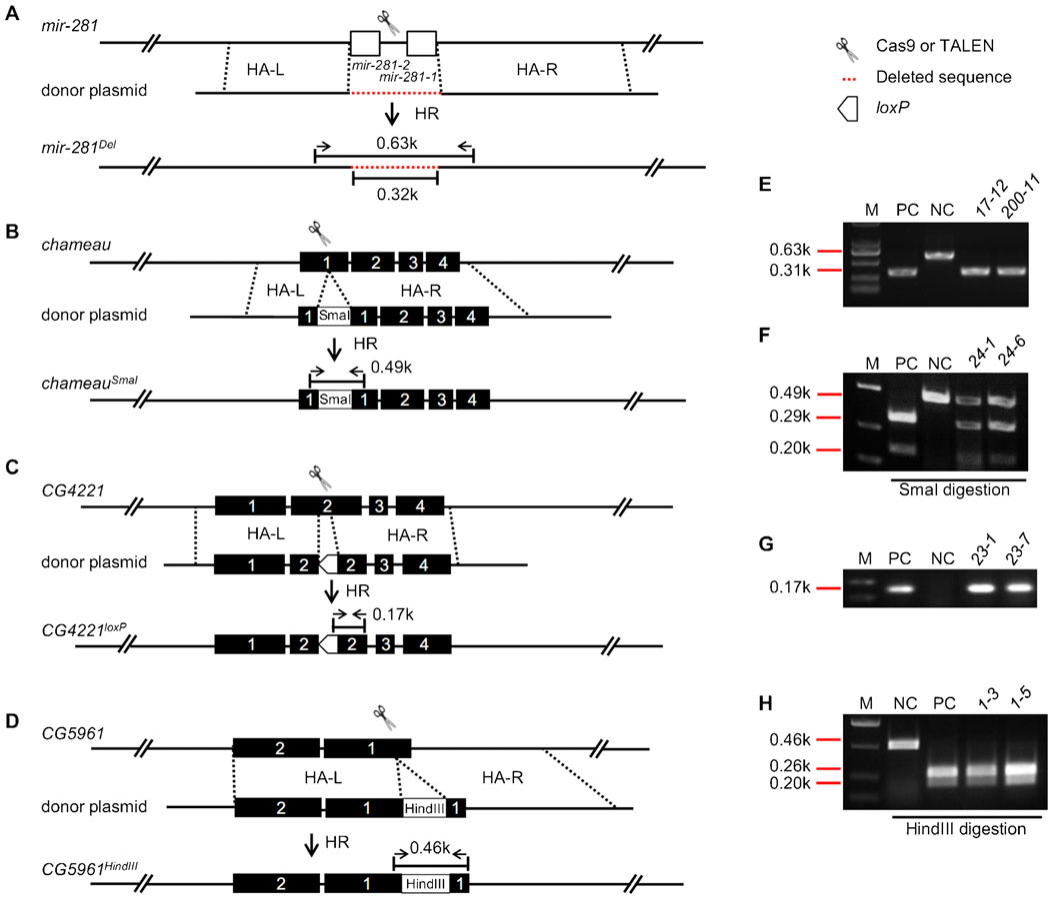
TALEN- and CRISPR/Cas9-mediated precise genomic deletion and nucleotides replacement. (A,E) TALEN-mediated *miR-281* deletion and molecular identification. (A) The pair of scissors indicates where the TALENs cut at the *miR-281* locus. Dashed red line indicates the deleted genomic region (0.32 kb). (E) The genomic DNAs of two homozygous lines, *17-12* and *200-11*, were used as PCR templates. Arrows in opposite directions indicate where the primers are located. The appearance of a 0.31 kb PCR product indicates successful deletion. (B,F) TALEN-mediated SmaI replacement at the *chameau* locus and molecular identification of positive events. (B) The pair of scissors indicates where the TALENs cut at the *chameau* locus. (F) Genomic DNAs of two heterozygous F_1_ lines, *24-1* and *24-6*, were used to show PCR and positive SmaI digestion results. Arrows in opposite directions indicate where the primers used for PCR are located (B). PCR products were digested by SmaI; the two cleaved fragments, 0.29 kb and 0.2 kb, represent successful nucleotide replacements. (C,G) CRISPR/Cas9-mediated *loxP* replacement at the *CG4221* locus and molecular characterization. (C) The scissors indicate where the CRISPR/Cas9 cleaves at the *CG4221* locus. The empty pentagon box represents the *loxP* site. (G) Genomic DNAs of two heterozygous F_1_ lines, *23-1* and *23-7*, were used for PCR examination. The primers are indicated by the two opposite arrows in panel C. The appearance of a 0.17 kb band indicates successful *loxP* replacement. (D,H) CRISPR/Cas9-mediated HindIII replacement at *CG5961* and molecular characterization. (D) The scissors indicate where the CRISPR/Cas9 cleaves at the *CG5961* locus. (H) Genomic DNAs of two homozygous F_1_ lines, *1-3* and *1-5*, were used for PCR and following HindIII enzyme digestions. The primers for PCR are indicated by the two opposite arrows in panel D. The appearance of two cleaved fragments, 0.26 kb and 0.20 kb, indicates successful HindIII replacement. M: DNA marker; NC: negative control, the corresponding PCR products were amplified from *Lig4^169^* genomic DNA with particular primers in each case. PC: positive control, the corresponding PCR products were amplified from the donor plasmid DNAs with particular primers in each case; k: kilo-base pair; HA-L and HA-R: left homologous arm and right homologous arm; HR: homologous recombination; donor plasmid: circular donor plasmid containing HA-L and HA-R and additional elements if any, the pBSK vector backbone is omitted here; all the dashed black lines indicate the homologous regions in the fly genome and on the donor plasmid; the empty boxes in panel A indicate the genomic regions of *miR-281*; all the filled boxes with numbers indicate the sequential coding sequences of the corresponding genes in each case, not necessarily representing the full coding sequences.

In the case of *miR-281*, a pair of TALENs (see figure legends and supplementary material Table S1 for details) was designed to generate a DSB within the *miR-281* loci (Fig. 1A). One pair of homologous arms (HAs) was selected from the flanking genomic regions of the *miR-281* loci (as indicated by HA-L, 1.3 kb, and HA-R, 1.9 kb, in Fig. 1A; supplementary material Table S3) and cloned into the pBSK vector to generate the donor plasmid that will be used to mediate the HDR. We expected co-injection of the donor plasmid and the TALEN mRNAs for *miR-281* into the *Lig4^169^* embryos would precisely delete the genomic DNA segment of both *miR-281-1* and *miR-281-2*. Indeed, three *miR-281* deletion-yielding F_0_ flies were identified from 65 total F_0_ flies, and four F_1_ flies were obtained from a total of 520 F_1_ flies, as determined by the appearance of a shorter PCR fragment (0.31 kb, 0.32 kb deleted) compared to that of the wild type (0.63 kb) (Fig. 1A,E; supplementary material Table S4). Two homozygous lines, *17-12* and *200-11*, were established and used for further confirmation of the short PCR fragment and for sequencing (Fig. 1E; supplementary material Fig. S1).

In the case of *chameau*, a pair of TALENs (see figure legends and supplementary material Table S1 for details) was designed for generating DSBs within the *chameau* loci (Fig. 1B). A *Sma*I restriction site was designed to replace an array of 13 nucleotides in the first coding exon of *chameau* in the donor plasmid, manipulation of which leads to a frame shift after the homologous arms (HA-L, 1.0 kb, and HA-R, 2.2 kb) are recombined with the endogenous DNA segments (Fig. 1B; supplementary material Table S3; Fig. S2). A pair of primers (Fig. 1B; supplementary material Table S4) was designed to generate PCR products that cover the SmaI site if the HDR events have occurred. After *Sma*I digestion, two fragments with the sizes of 0.29 kb and 0.20 kb should indicate the HDR events (Fig. 1B,F). Five F_1_ mutant alleles of *chameau* that had a precise *Sma*I site insertion were identified among 363 total F_1_ offspring from three independent F_0_ lines. Further sequencing was performed to confirm the precise replacement (supplementary material Fig. S2).

The statistics of both the precise deletion of *miR-281* and precise DNA replacement in *chameau* are summarized in Table 1. Collectively, we have successfully generated two precise genomic modifications in *Drosophila* through TALEN induced DSBs and exogenous donor plasmids, which induced the HDR pathway. The frequency of yielding inheritable HDR modifications ranged from 0.8% to 1.4% in F_1_ flies, and the frequency of homologous recombination (HR) yielding F_0_ in total fertile F_0_ ranged from 4.6% to 5.8% in F_0_ flies.

**Table 1.**
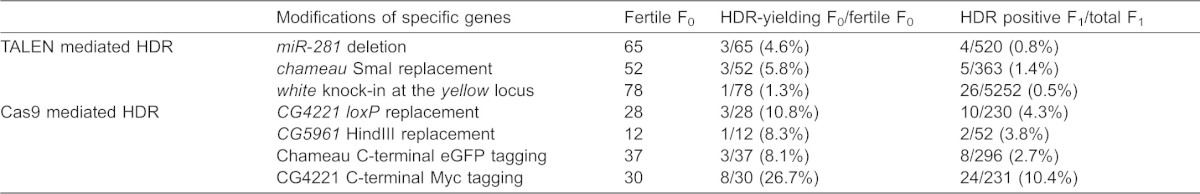
Summary of HDR frequencies mediated by TALEN or CRISPR/Cas9 at different loci

### CRISPR/Cas9-mediated precise mutagenesis via the HDR pathway

Our results described above indicate that the efficiency of TALEN-mediated HDR is relatively low as expected, significantly lower than the efficiency to generate TALEN-mediated indels (Liu et al., 2012). The CRISPR/Cas9 system has been shown to be more efficient than TALEN in mediating genomic indel mutations (Yang, L. et al., 2013). We wondered whether the CRISPR/Cas9 system would also be more efficient in mediating HDR in *Drosophila*. For this purpose, we chose two *Drosophila* genomic loci, *CG4221* and *CG5961*, to conduct HR-mediated mutagenesis using the CRISPR/Cas9 system. No mutant alleles have been characterized thus far for either *CG4221* or *CG5961*.

In the case of *CG4221* mutagenesis, we used a *loxP* site to precisely replace the putative 0.12 kb F-box domain of *CG4221* (Dui et al., 2012) (Fig. 1C; supplementary material Fig. S3), leading to a disruption of the gene's coding function. The *loxP* site can be used for the removal of the unwanted “ends-in” recombination (see Results, last section). The donor plasmid that contains the HA-L (1.5 kb) and HA-R (1.3 kb) (Fig. 1C), and the designed Cas9 mRNA/gRNA (Fig. 1C; supplementary material Table S2, Table S3), were co-injected into *Lig4^169^* embryos to induce HDRs. Successful replacement was detected in 10 out of 230 F_1_ flies, as assayed by the positive PCR product yielded with primers that reside in the *loxP* site and the coding exon 2 (Fig. 1C,G; supplementary material Fig. S3; Table S4).

In the case of *CG5961* mutagenesis, the strategy was similar to that used for *chameau* mutagenesis. A HindIII restriction site was designed between two homologous arms (HA-L, 1.3 kb, and HA-R, 1.4 kb) in the donor plasmid (Fig. 1D; supplementary material Fig. S4; Table S2, Table S3) to replace 40 bp of endogenous DNA in the first coding exon of *CG5961*. Successful replacement of the endogenous DNA with the HindIII site generated a 0.46 kb band that was amplified with two primers as indicated in Fig. 1D, from which two bands were yielded following HindIII enzyme digestion of the PCR products (Fig. 1H). Two mutant F_1_ flies that showed positive HindIII digestion were obtained from 52 F_1_ flies.

We have successfully targeted two genomic loci via the CRISPR/Cas9-induced HDR pathway. These results indicate the frequency of inheritable precise mutagenesis induced by CRISPR/Cas9-mediated HDR seems to be higher than that of TALEN, ranging from 3.8% to 4.3%, as is also the case of HR yielding F_0_/total fertile F_0_ as shown in Table 1, ranging from 8.3% to 10.8%. These observations were also consistent with reports that CRISPR/Cas9 is generally more efficient in mediating normal indels from the non-homologous end joining (NHEJ) pathway (Liu et al., 2012; Yu et al., 2013a).

### Comparison of TALEN- and CRISPR/Cas9-mediated HDR at the same chromosomal region of the *yellow* locus

**Fig. 2.**
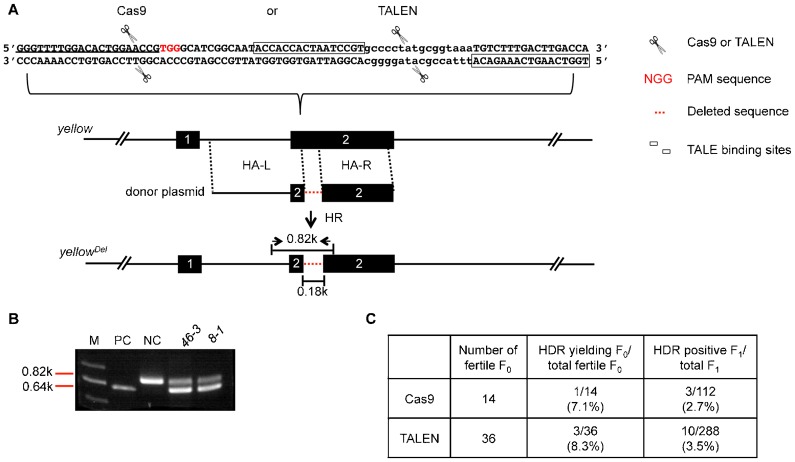
Comparison of TALEN- and CRISPR/Cas9-mediated HDR at the *yellow* locus. (A) CRISPR/Cas9- or TALEN-mediated *yellow* deletion. The CRISPR/Cas9 binding sequence is 5′-GGGTTTTGGACACTGGAACCG-3′ (underlined in panel A); PAM sequence is marked in red. One pair of TALEN binding sites is marked by boxes. The pairs of scissors indicate the Cas9 or TALEN cutting site at the *yellow* locus. The dashed red lines represent the deleted *yellow* genomic sequence (0.18 kb). (B) Molecular identification of the *yellow* deletions. The genomic DNAs of two heterozygous F_1_ lines, *46-3* (CRISPR/Cas9-mediated mutagenesis) and *8-1* (TALEN-mediated mutagenesis) were used as examples. The pair of primers used for PCR is shown in panel A (opposing arrows). The appearance of a 0.46 kb PCR band indicates successful deletion. (C) Frequencies of CRISPR/Cas9- and TALEN-mediated HDR at the *yellow* locus. The deletion-yielding events in both F_0_ and F_1_ are scored based on the appearance of the 0.64 kb short PCR product shown in panel B. The legends for the rest of the elements/labels are the same as in Fig. 1.

Although a generally higher efficiency of CRISPR/Cas9-induced HDR (3.8–4.3%) was observed compared with TALEN-induced HDR (0.8–1.5%), conclusions cannot be made before the following possibilities are excluded: more accessible local chromatin structure or different donor sequences. To investigate these possibilities, we set out to further compare the efficiency of TALEN- and CRISPR/Cas9-induced HDR at the same *yellow* locus using the same exogenous donor (Fig. 2A; supplementary material Fig. S5; Tables S1–S3). Here, both the TALENs and the gRNA for the CRISPR/Cas9 have been shown to efficiently induce NHEJ-mediated indels as previously described (Liu et al., 2012; Yu et al., 2013a). The exogenous donor plasmid was designed to delete both the TALENs and the gRNA recognition sequences (boxed for the TALENs and underlined for the gRNA), between HA-L (1.9 kb) and HA-R (1.1 kb), at the *yellow* locus, which generate a 0.18 kb shorter PCR product than *Lig4^169^* flies (from 0.82 kb to 0.64 kb) with the indicated primers (Fig. 2A,B; supplementary material Fig. S5). This PCR strategy was employed to determine whether any HDR events have occurred after TALEN- or CRISPR/Cas9-induced DSBs. Interestingly, statistical analysis showed the ratio of detected HDR events in F_1_ was 2.7% for CRISPR/Cas9, whereas the ratio was 3.5% for TALEN; similar results were obtained for mutation-yielding F_0_/total fertile F_0_, which was 7.1% versus 8.3% (Fig. 2C). These results suggest that TALENs are likely as efficient as CRISPR/Cas9 to induce HDRs in the *Drosophila* genome, at least at the *yellow* locus.

### *In vivo* tagging through CRISPR/Cas9-induced homologous recombination

After showing that both TALEN and CRISPR/Cas9 can efficiently mediate precise genomic modifications such as deletions and nucleotides replacement, we set out to explore precise insertions. The best example for precise genomic insertions is *in vivo* tagging, which can help to solve several technical problems in biological research such as: (1) Antibodies are widely considered as one of the most important tools to dissect functions of specific genes. However, due to the high cost, low success rate and time-consuming labor, it is still a problem to get ideal antibodies for many proteins; (2) Transgenes with tags often exhibit ectopic expression, whereas *in vivo* tagging leads to the precise tracing of endogenous protein expression; and (3) Proteins with fluorescent tags can be used in live imaging, which is an essential technology for developmental biologists. Here, we thought to take the advantage of HDRs induced by the highly efficient CRISPR/Cas9 technology to tag two endogenous proteins, Chameau and CG4221, with two tags of eGFP (0.72 kb) and Myc (less than 0.1 kb), respectively, that are in different sizes.

**Fig. 3.**
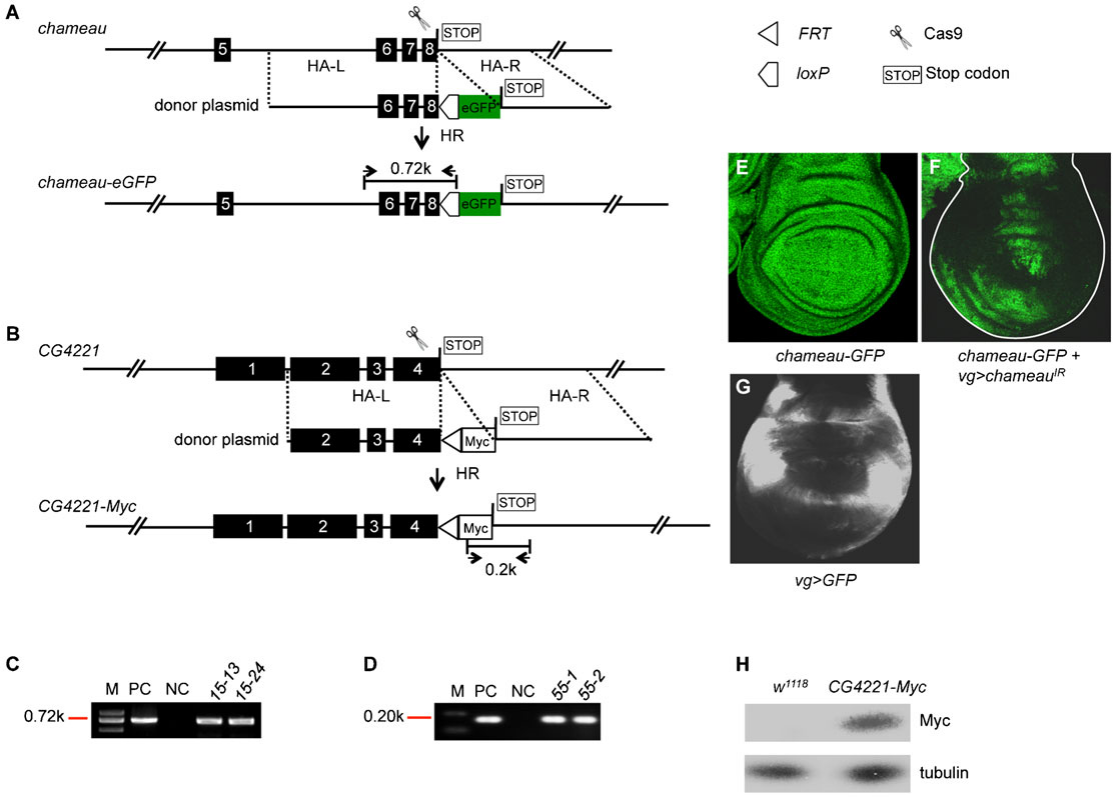
*In vivo* tagging at the *chameau* and *CG4221* loci. (A,C) *chameau* C-terminal *eGFP* tagging. (A) The cartoon scissors represent the CRISPR/Cas9 and indicate where it cuts at the *chameau* locus. The empty pentagon box indicates *loxP* site. The filled green box marked with eGFP indicates where the *eGFP* fragment is fused. The empty box marked by STOP indicates the stop codon of the *chameau* gene. (C) The genomic DNAs of two heterozygous F_1_ lines, *15-13* and *15-24* were used for PCR templates. The pair of primers used for PCR is shown in panel A as the two opposing arrows. The appearance of 0.72 kb PCR products indicates successful eGFP tagging (precise insertions). (B,D) *CG4221* C-terminal *Myc* tagging. (B) The cartoon scissors indicate the Cas9 nuclease and its cutting site at the *CG4221* locus. The empty triangle indicates the *FRT* site. The empty box marked with Myc indicates the *Myc* sequence. The empty box marked by STOP represents the stop codon of the gene *CG4221*. (D) The genomic DNAs of two heterozygous F_1_ lines, *55-1* and *55-2* were used as templates for PCR detection. The pair of primers used for PCR is shown as the two opposing arrows in panel B. The positive 0.2 kb PCR band indicates successful Myc tagging (precise insertions). (E–G) Immunostaining detection of the expression of Chameau-eGFP. (E) 3^rd^ instar larval wing discs of line *15-13* were stained with the anti-eGFP antibody. (F) Knockdown of *chameau* by *vg>chameau^IR^* led to loss of GFP signals in the *vg-Gal4* expression regions. The genotype in panel F is *w; chameau-eGFP/vg-Gal4; chameau^IR^/+*. The wing disc boundary is marked by a dotted white line in panel F. (G) Expression pattern of *vg-Gal4* in 3^rd^ instar larval wing discs. *vg>GFP*: *w; vg-Gal4/UAS-eGFP*. GFP: green fluorescent protein; eGFP: enhanced green fluorescent protein. (H) Detection of the expression of CG4221-Myc fusion protein by Western blot. Embryos of *w^1118^* and *CG4221-Myc* line *55-1* were collected for Western blot at 0–3 hours. Anti-Myc antibodies were used to detect CG4221-Myc and tubulin was used as a loading control. See Fig. 1 legend for the common elements or labels that are not explained here.

For *in vivo* tagging of the *chameau* gene, we wished to insert an enhanced *GFP* (*eGFP*) sequence before the stop codon, resulting in a fused gene, *chameau-eGFP* (Fig. 3A; supplementary material Fig. S6). The donor plasmid that contains the *eGFP* sequence between two homologous arms (HA-L, 2.8 kb and HA-R, 1.8 kb) and the Cas9 mRNA/gRNA for generating a DSB before the stop codon of *chameau* coding sequence were co-injected into *Lig4^169^* embryos (Fig. 3A; supplementary material Tables S2, S3). Successful insertion of *eGFP* was detected by PCR using primers embedded in the inserted sequence and genomic sequences (Fig. 3A,C; supplementary material Fig. S6). RT-PCR was also employed to detect the *chameau-eGFP* transcripts (supplementary material Fig. S7A,B; Table S4). Expression validation of the Chameau-eGFP protein was performed by immunostaining of eGFP in the wing imaginal discs of 3^rd^ instar larvae. As shown in Fig. 3E, GFP is ubiquitously expressed in the wing imaginal discs of line *15-13*, manifesting the pattern of endogenous Chameau. To further demonstrate the existence of the fused *chameau-eGFP* transcripts, we used *vestigial-Gal4* (*vg-Gal4*) to drive *UAS-chameau^RNAi^* to knockdown the transcription of *chameau* at the *vg-Gal4* expressing regions (Fig. 3F,G). Given *chameau* and *eGFP* formed a fusion transcript, when *chameau* was knocked down, the expression of GFP would also be expected to be reduced in the corresponding regions of *chameau* RNAi. The staining results shown in Fig. 3F indicate that GFP signals were significantly down-regulated where *vg-Gal4* driven *chameau^RNAi^* was present. Collectively, these results indicate *eGFP* was successfully inserted into the C-terminus of the gene *chameau*, leading to a fusion protein that can be detected by the anti-GFP antibody. Since the flies carrying the Chameau-eGFP fusion protein did not exhibit any abnormality, we predict the fusion protein functions normally *in vivo*.

For *in vivo* tagging of the *CG4221* gene, we wished to insert a Myc tag before the stop codon, resulting in a fused gene: *CG4221-Myc*. The donor plasmid that contains the Myc sequence and an *FRT* site (see Discussion) between two homologous arms (HA-L, 1.6 kb and HA-R, 1.8 kb), and the corresponding Cas9 mRNA/gRNA, were again co-injected to the *Lig4^169^* embryos (Fig. 3B; supplementary material Fig. S8; Tables S2, S3). F_1_ flies that carry successful Myc insertions were screened by PCR using a primer embedded in the Myc sequence (Fig. 3B; supplementary material Table S4). Two positive PCR products from lines *55-1* and *55-2* are shown in Fig. 3D. PCR using primers outside of the homologous arms and RT-PCR were performed to further confirm the insertion and expression (supplementary material Fig. S9; Table S4). To further validate the expression of CG4221-Myc protein, we employed Western blot to detect the Myc tag. As shown in Fig. 3H, a Myc-band corresponding to the size of CG4221-Myc protein was detected from total embryo lysates of line *55-1* using an anti-Myc antibody, whereas this band was missing in wild-type control embryos.

We have tagged two endogenous genes with two different markers, eGFP and Myc, respectively, via the CRISPR/Cas9-mediated HDR pathway. The efficiency of these kinds of *in vivo* tagging was sufficiently high for practical utilization. Strikingly, for a 0.72 kb *eGFP* tagging, the efficiency of getting inheritable germline insertions was 2.7%; and for a smaller tag of Myc the germline efficiency was as high as 10.4%. The summary of the statistical results is shown in Table 1.

### The *white* platform for an easy screen of TALEN- or CRISPR/Cas9-mediated genome-wide mutagenesis

**Fig. 4.**
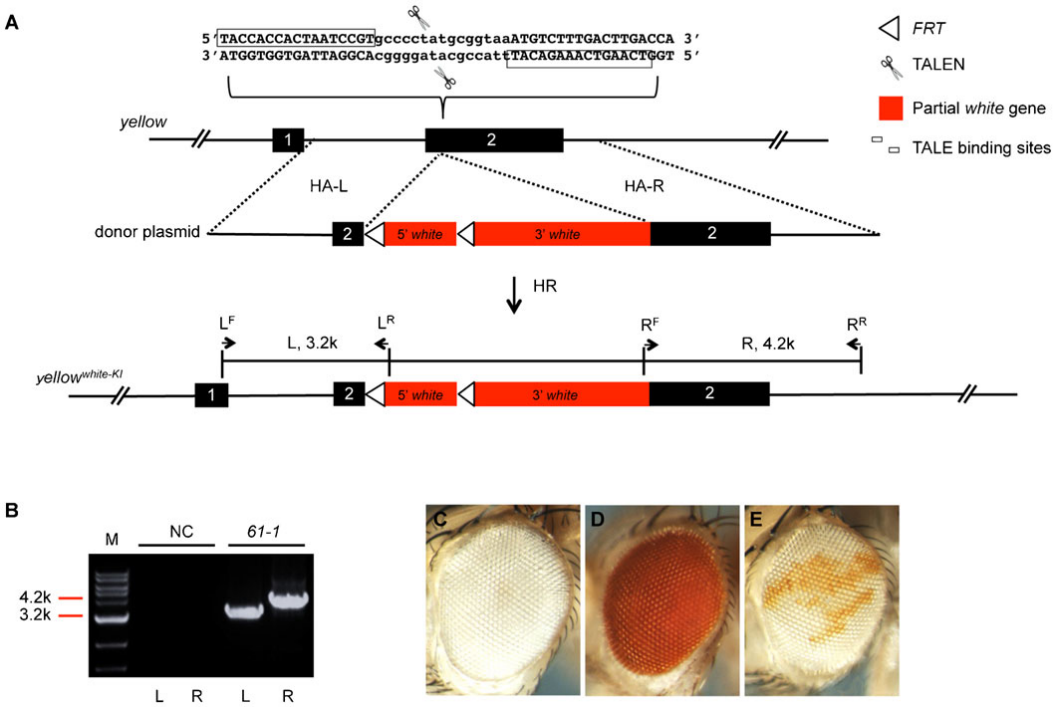
The easy-to-screen *white* platform for the *yellow* mutagenesis. (A) Schematic representation of the generation of HDR-mediated *yellow* mutagenesis using the easy-to-screen *white* platform. The cartoon scissors indicate the TALENs and their cutting site at the *yellow* locus. The pair of TALEN binding sites is marked by the boxes. The empty triangles are two *FRT* sites located in the 5′ regulatory region and the intron of the *white* gene, and are oriented in the same direction. The two red-filled boxes indicate two genomic parts of the *white* gene separated by the second *FRT* site. Arrows with the names of L^F^, L^R^, R^F^, R^R^ indicate the primers used for PCRs to get the L (left) and R (right) fragments as indicated in panel B. (B) Molecular identification of the *white* knock-in at the *yellow* locus, resulting in a simultaneous mutation in *yellow*. The genomic DNA of heterozygous F_1_ line, *61-1*, was used for showing the positive PCR results. L^F^ and L^R^ were used as primers to get the L fragment. R^F^ and R^R^ were used as primers to get the R fragment. (C–E) Removal of the *white^+^* marker carried in the *yellow* mutants. (C) An eye of *Lig4^169^* flies. (D) An eye of the *yellow* mutant line, *61-1*, which carried the *white* knock-in. (E) A mosaic *white* eye phenotype induced by heat-shock on the offspring of line *61-1* that was crossed with *hs-Flp*. See Fig. 1 legend for the common elements or labels not explained here.

We have shown both TALEN and CRISPR/Cas9 systems are efficient for modifying the fly genome via the HDR pathway. However, it is still relatively time-, labor-, and money-consuming when using PCR based screening strategies to search for correctly modified genomes. Here, we chose to develop an easy-to-screen system using the *Drosophila white* gene as a marker. In order to set up this system, we first modified the pP[RS3] plasmid (Golic and Golic, 1996) to be a vector that contains the *white* coding sequence and its minimum regulatory sequence between two multiple cloning sites (MCS) (see Materials and Methods for more details; supplementary material Fig. S10). The specific elements of this vector include: (1) 5′ and 3′ MCSs flanking the *white* region for the insertion of different homologous arms (HA-L, 3.2 kb and HA-R, 4.2 kb); and (2) two *FRT* sites (5′*FRT* and 3′*FRT* in the same direction) located in the 5′ regulatory region and the intron of the *white* gene, respectively. The FRTs can be used for further removal of *white* expression by the Flp recombinase (see Materials and Methods; Fig. 4A; supplementary material Fig. S10). The modified vector was designated pP[RS3]^3′M^.

To test how this system works for targeted mutagenesis through the HDR pathway, we employed the same TALENs (supplementary material Table S1) used in Fig. 2A to induce a DSB at the *yellow* locus. The donor plasmid, pP[RS]^3′M^-yellow^white-KI^, was designed to contain homologous arms (HA-L and HA-R in Fig. 4A) of the *yellow* gene that flank the *white* gene in pP[RS3]^3′M^ (see Materials and Methods; Fig. 4A). After co-injection of the TALENs and the donor DNAs, successful mutagenesis of the *yellow* gene was simply screened by following the *white*^+^ eye phenotype (red eyes), which simultaneously expresses the *yellow* mutation, leading to *yellow* phenotypes of the red-eyed flies (Fig. 4D). PCR analysis was used to further confirm the *white* knock-in (KI) using two pairs of primers (L^F^ and L^R^, R^F^ and R^R^), as indicated in Fig. 4A, to yield two positive bands of 3.2 kb and 4.2 kb (Fig. 4B). The efficiency of obtaining the *yellow^white-KI^* flies was 0.5% in germline transmission, and the frequency of HR yielding F_0_ in total fertile F_0_ was 1.3% as shown in Table 1.

Mutant flies created in this way will carry the *white*^+^ marker, which can be useful for neurobiologists performing visual behavior experiments, but might be unwanted due to differences in genetic backgrounds for other tests. To remove the *white^+^* marker, the two *FRT* sites that flank the 5′ part of the *white* gene are used (Fig. 4A). Such mutant flies were crossed with flies carrying the *hs-Flp* transgene. After a brief heat shock at 37°C, the mosaic *white* phenotype was observed in offspring flies, indicating the *hs-Flp* was efficient in removing the *FRT* cassettes that reside in the *yellow* mutants (Fig. 4E). With this easy-to-screen *white* platform, in principle, one can generate (screen for) any precise genomic mutations using only binocular microscopes without high-throughput PCR and enzyme digestion.

### Removal of “ends-in” recombination events

**Fig. 5.**
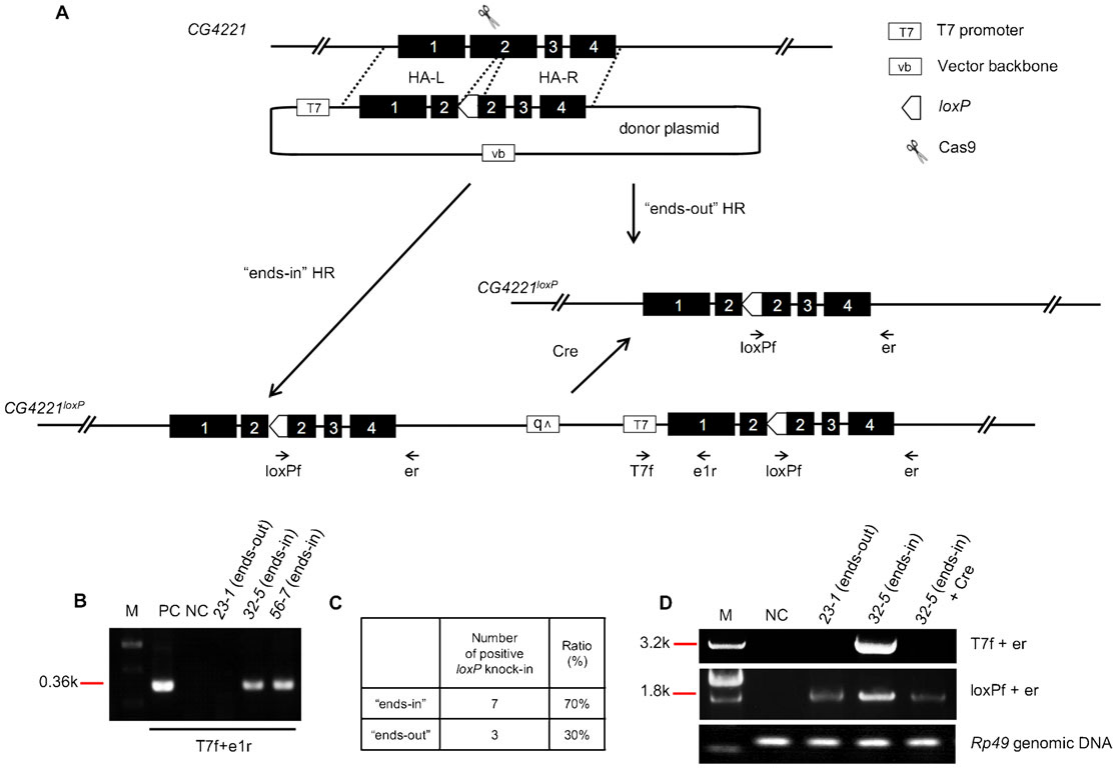
Removal of “ends-in” recombination resultant from *CG4221* mutagenesis. (A) “Ends-in” and “ends-out” homologous recombination (HR) were generated via the HDR pathway in the process of *CG4221* mutagenesis, leading to either two copies of the *CG4221* mutations or one copy of the *CG4221* mutation in the fly genome, respectively (see also supplementary material Fig. S11). The Cre recombinase was introduced into the “ends-in” line to remove the duplicated copy of *CG4221* by flipping out the DNAs between the two *loxP* sites, converting the “ends-in” into the “ends-out” events. The short arrows with names indicate the primers used in panels B,D. (B) PCR assays to distinguish the “ends-in” and “ends-out” HR events occurred during *CG4221* mutagenesis. The genomic DNAs of heterozygous F_1_ lines *23-1*, *32-5* and *56-7* were used for PCR assays. T7f and e1r detect a corresponding 0.36 kb “ends-in” band, while nothing for the “ends-out”. (C) “Ends-in” and “ends-out” HR ratio of *CG4221* mutagenesis, based on the PCR analyses as described in panel B. (D) Molecular confirmation of the removal of the “ends-in” products. Line *32-5* was selected as an example to show successful removal of additional sequences between the two *loxP* sites by Cre recombinase, as indicated by the loss of the band amplified with T7f and er. Primer pairs used for PCRs are indicated on the right of the gel. *Rp49* genomic primers were used for genomic DNA quality control. See the legends to Fig. 1 for the common elements or labels that are not explained here.

In the process of precise modifications of the *Drosophila* genome through the HDR pathway by co-injection of a circular homologous donor, two ways of holiday-junction resolution lead to two kinds of outcome: “ends-out” and “ends-in” (Fig. 5; supplementary material Fig. S11). The “ends-in” recombination, as in the case of *chameau* mutagenesis (supplementary material Fig. S12), might be problematic depending on the purpose of the genomic modifications. When the purpose is to mutate a genomic locus, and the “ends-in” recombination yields two mutant alleles at the same locus, it is not a concern for phenotypic analyses. However, if the purpose were to tag an endogenous gene or to make gene corrections, a clean “ends-out” recombination would be favorable. The frequency of getting “ends-in” events can vary from 0 to 100% according to what we observed in this study (Fig. 5C; supplementary material Fig. S12C; data not shown), likely depending on the genomic locus and donor plasmid design, particularly, the distance between the DSB site and the proximal ends of the homologous arms.

Rong and colleagues reported a reduction strategy to remove a duplicated copy resulting from the ends-in recombination (Rong et al., 2002). Here we offer a different solution to remove the “ends-in” recombination and “convert” this kind of event into the “ends-out” outcome, taking advantage of the Flp-*FRT* or Cre-*loxP* systems. The *CG4221* mutagenesis case will be used again to explain how the system works (Fig. 5). When the HDR pathway is triggered by the presence of both a DSB and a circular homologous donor, two forms of HDR products will be generated (Fig. 5; supplementary material Fig. S11). The “ends-in” HDR event results in a genomic duplication of the targeted *CG4221*, separated by the vector backbone (vb) of the donor plasmid, whereas the “ends-out” HDR yields only a mutated *CG4221* locus (Fig. 5A). To molecularly distinguish these two events, two PCR strategies were again used. A quick examination expects a short PCR product for the “ends-in” event, but not for the “ends-out” event, using one primer located in the T7 of the vector bone (T7f) and another in the first exon (e1r) (Fig. 5A; supplementary material Table S4). Indeed, a PCR product of 0.36 kb was observed in the “ends-in” recombination, but not in the “ends-out” case (Fig. 5B). For further confirmation, a longer, 3.18 kb PCR product extending from the T7f primer to the e1r primer located in the 3′ region of *CG4221* locus was obtained from the “ends-in” flies. The longer PCR product disappeared in the same flies when the Cre recombinase was introduced to remove the sequences between two *loxP* sites, leaving a clean mutation resembling that from the “ends-out” event (Fig. 5A,D).

These results indicate that, introducing a *loxP* site into the homologous donor to be recombined into the to-be-modified locus of the fly genome, together with a Cre recombinase, can remove the “ends-in” recombination by “converting” the “ends-in” outcome into an “ends-out” outcome, when needed.

## DISCUSSION

In recent years, genome editing technologies such as the zinc finger nuclease (ZFN), TALEN and CRISPR/Cas9 have emerged to facilitate biomedical research including targeted gene modifications in both basic theoretical research and applied research such as cancer studies (de Souza, 2012; Beumer et al., 2013; Piganeau et al., 2013; Wei et al., 2013). Among them, TALEN and CRISPR/Cas9 systems have been quickly adopted by biologists due to their high efficiencies in DSB induction and easy-to-handle procedures. However, most of the studies thus far have focused on using these two systems in different organisms to generate indel (insertion and/or deletion) mutations via the NHEJ repair pathway or to generate targeted insertion via homology-independent repair (Auer et al., 2014; Maresca et al., 2013; Wei et al., 2013). Despite recent reports of TALEN- or CRISPR/Cas9- mediated HDR in culture cells (Bassett et al., 2014; Yang, H. et al., 2013), application in model organisms has been, thus far, poorly explored. HDR-based genomic modifications include basically three types: (1) precise controllable deletions, which can be applied for deleting specific functional domains of particular genes, or generating precise null alleles of non-coding RNAs or genes with multiple splicing variants; (2) nucleotide replacements, which can be employed for gene corrections and precise point mutations; and (3) insertions, which can be used for *in vivo* tagging of protein-encoding genes or generating duplicating genes. In this study, we used *Drosophila* as the model organism to test the applications of HDR-mediated genomic modifications through TALEN- and CRISPR/Cas9-based systems. We successfully generated precise deletions, nucleotide replacements and insertions with a germline transmission efficiency of up to 10.4%; we compared the efficiencies of TALEN- and CRISPR/Cas9-mediated HDRs; we established an easy-to-screen platform for precise mutagenesis through TALEN- and CRISPR/Cas9-mediated HDRs; and last but not least, we offered a strategy to resolve the “ends-in” recombination resulting from co-injection of a circular homologous donor. Our results and related tools will be very helpful in facilitating *in vivo* genomic engineering in *Drosophila* as well as *in vivo* functional studies.

Based on our statistical results (Fig. 2C; Table 1), the F_1_ efficiency of CRISPR/Cas9-mediated genomic modifications via the HDR pathway was between 2.7% and 10.4%, which seemed to be higher than that of TALEN (0.5% to 3.5%). These results suggested CRISPR/Cas9 may be generally more efficient than TALEN in inducing the HDR pathway in *Drosophila*. In principle, this could be due to a higher efficiency in generating DSBs at targeted loci, which has also been supported by recent studies in several different organisms using either CRISPR/Cas9 or TALENs in inducing NHEJ-mediated indel mutations (Liu et al., 2012; Sung et al., 2013; Wang et al., 2013; Yang, L. et al., 2013; Yu et al., 2013a). However, a direct comparison of efficiencies between CRISPR/Cas9- and TALEN-mediated genomic modifications is still lacking both *in vivo* and *in vitro*. To directly compare the efficiency of TALEN and CRISPR/Cas9 in mediating HDRs, we forced these two enzymes to act on the same locus of *yellow* (Fig. 2) in the presence of the same donor. Our results suggest the efficiency of TALEN actually was comparable to that of CRISPR/Cas9 in mediating HDRs in *Drosophila*. More cases may be needed to reach a definite conclusion. On the other hand, in all our cases of HDR-mediated applications either by TALEN or by CRISPR/Cas9, the efficiencies seemed to be high enough for practical manipulations in the lab; even in the case of 5 kb *white* knock-in (Fig. 4; Table 1), the efficiency was still as high as 0.5% in F_1_ flies. Nevertheless, the CRISPR/Cas9 system should be more convenient in RNA preparations than the TALEN.

NHEJ and HDR are two major pathways to repair DSBs. In this study, we focused on investigations of TALEN- or CRISPR/Cas9-induced HDR in the presence of homologous donor plasmids. To promote HDR versus NHEJ events in all the cases, a *Ligase4* mutant (*Lig4^169^*) was employed. However, in the case of *yellow* deletion, only 20.0% (19/95) and 20.0% (8/40) HDR events were observed in both TALEN- and CRISPR/Cas9-induced *yellow* mutant F_1_, respectively. 80.0% of the *yellow* F_1_ in each case were non-HDR mutants (as assayed by PCR). Notably, this ratio of HDR versus non-HDR events was apparently lower than that in the case of ZFN-induced HDR (99.1%) at the *rosy* locus reported by Beumer and colleagues (Beumer et al., 2008).

“Ends-in” and “ends-out” are two basic recombination events (outcomes) of HDR in the presence of a genomic DSB and a circular homologous donor plasmid. We detected these two outcomes of recombination in some of our mutagenesis cases: *chameau* SmaI replacement (supplementary material Fig. S12) and *CG4221 loxP* replacement (Fig. 5; supplementary material Fig. S11). To remove the unwanted duplication resultant from the “ends-in” recombination, we took advantage of the *Cr*e*/loxP* system using *CG4221* mutagenesis as an example (Fig. 5). Statistical results indicated that 70% of F_1_ alleles were “ends-in” recombinations in this particular case (Fig. 5B), whereas in *chameau* SmaI replacement, the “ends-in” recombinations comprised 100% (supplementary material Fig. S12). Now the question arises: what determines the “ends-in” versus “ends-out” ratio? Although our study has not addressed this question in detail, we believe that the design of the donor plasmid, the relative distance between the proximal ends of the homologous sequences and the cutting site, and the length in between the two homologous arms may play essential roles in determining the outcome of “ends-in” and “ends-out” events. Further systematic experiments are needed to eventually answer this question.

In our study, two different strategies were employed to ensure that the TALEN- and CRISPR/Cas9-mediated HDR events occurred at the target loci: 1) PCR-based molecular analysis using a combination of internal and external primers (supplementary material Fig. S7A,C, Fig. S9A,C, Fig. S12A,B); and 2) expression detection of the fusion protein Chameau-GFP, which is expected to be under the control of the endogenous promoter of the *chameau* gene (Fig. 3). However, possible off-target events cannot be totally excluded at this stage, since technical difficulties in some of the cases, only internal primers, which were embedded in the donor homologous sequences, were used for PCR detections.

## MATERIALS AND METHODS

### Fly stocks

All flies used in this study were obtained from the Bloomington Stock Center and cultured at 25°C unless otherwise stated. Genotypes of these flies are as follows: *w^1118^,* *Lig4^169^* (BL28877); *w^1118^, P{70FLP}10* (BL6938); yw, P{Cre,y^+^}1b; D*/TM3, Sb (BL851); *w, vestigial-Gal4; TM2/TM6B, Tb* (vg-Gal4, BL6819); *yv, P{TRip.HMS00487}attP2* (UAS-chameau^RNAi^, BL32484).

### Design of TALENs, gRNAs and donor plasmids

The target DNA sequences for the left and right TALEs to bind and the spacer DNA in between were designed using the TAL Effector Nucleotide Targeter software (Cermak et al., 2011; https://tale-nt.cac.cornell.edu). Rules for designing TALE repeats have been described in our previous study (Liu et al., 2012). All the target and spacer DNA sequences selected for this study are listed in supplementary material Table S1. The TALE repeats were constructed according to the “Unit Assembly” procedure described elsewhere (Huang et al., 2011).

Target DNA binding sequences for customized gRNAs in *Drosophila* were selected according to the rules described in our previous study (Yu et al., 2013a). To simplify the gRNA designing and subsequent *in vitro* transcription, we followed the target sequence principle: 5′GG-N_17–19_-NGG-3′. The sequences of gRNAs used in this study are listed in supplementary material Table S2.

The pP[RS]^3′M^ plasmid used for our easy-to-screen *white* platform was modified from pP[RS3] (Golic and Golic, 1996; Drosophila Genomics Resource Center, Indiana; supplementary material Fig. S10). Briefly, a fragment spanning from the 3′ end of the *white* gene to the 3′ P element was amplified from the pP[RS3] vector by standard PCR using one pair of primers: the forward primer, 5′-CTCAAATGGTTCGAGTGGT-3′ and the reverse primer, 5′-AAATTGTACAACGACGCGTCGAGGCGCGCCTGCGAGTACGCAAAGCTAATTCAT-3′. The PCR products cut with AscI and MluI restriction enzymes were inserted at the Bsp1407 site of pP[RS3] with the correct orientation, establishing the basis of the *white* platform. Mutagenesis of the *yellow* gene was employed to demonstrate how to use this *white* platform. The left (2.5 kb) and the right (3.4 kb) homologous arms (HA), obtained through PCR (Fig. 4A; supplementary material Table S3) with the *Lig4^169^* genomic DNA as the template were inserted into the KpnI/NotI and the AscI/MluI sites, respectively, of the pP[RS3]^3′M^ vector to make the donor plasmid, pP[RS3]3′M-yellow^white-KI^. The primers used for amplifying HAs are listed in supplementary material Table S3.

For the rest of the donor plasmids used in this study, pBluescript SK (pBSK) was chosen to be the cloning vector because of the convenience of its multiple cloning sites. Briefly, the left and the right homologous arms were amplified by standard PCR procedure before digested and inserted into the pBSK vector. Donors with correct HAs were chosen for subsequent microinjections. In addition to the HAs, a *loxP* sequence and a linker sequence were cloned between the HAs in *CG4221 loxP* replacement; a *loxP* sequence and an eGFP coding sequence were inserted before the stop codon of *chameau*; an *FRT* sequence and a *Myc* tag sequence were added at the 3′ end of *CG4221* prior to the stop codon. The primers used for each donor construction are listed in the supplementary material Table S3.

### *In vitro* syntheses of the TALENs, Cas9 and gRNAs

TALEN mRNAs and Cas9 mRNAs/gRNAs were transcribed *in vitro* according to previously published protocols (Liu et al., 2012; Yu et al., 2013a). Specifically, pCS2-TALEN-L, pCS2-TALEN-R and pSP6-2sNLS-spcas9 plasmids with correct insertions were linearized and recovered as corresponding templates. Transcriptions were carried out following the instructions of the Sp6 mMESSAGE mMACHINE Kit (Ambion, USA). For Cas9 *in vitro* transcription, the poly (A) signals were added to the 3′ end of the capped mRNAs by *E. coli* Poly(A) polymerase Kit (New England BioLabs, USA). For the *in vitro* transcription of customized gRNAs, the DNA templates were obtained from the pMD19-T gRNA scaffold vector by PCR (Yu et al., 2013a). The transcription was carried out using the RiboMAX Large Scale RNA Production Systems-T7 Kit (Promega, USA). Each pair of purified TALEN mRNAs and the corresponding donor plasmid were mixed to a final concentration of 500 ng/µl for the mRNA and 700 ng/µl for the donor DNA, respectively; purified Cas9 mRNA, gRNA and donor plasmid were mixed to a final concentration of 750 ng/µl for the mRNA, 10 ng/µl for the gRNA and 700 ng/µl for the donor DNA, respectively.

### Microinjection

The prepared injection mixtures were centrifuged at maximum speed before loading in needles for microinjection. Mixtures were injected into *w^1118^*, *Lig4^169^* embryos according to the standard procedure as previously described (Huang et al., 2010; Yu et al., 2013b).

### Screening and statistical analyses of mutations resulting from TALEN- and Cas9-mediated homologous recombination

For the cases of *miR-281* deletion, *CG4221* mutagenesis, Chameau C-terminal eGFP tagging and CG4221 C-terminal Myc tagging, the ratio of HR events in F_0_ was calculated based on single crosses that yielded inheritable HR F_1_. The ratio of HR in F_1_ was calculated based on PCR results of F_1_ flies, which were picked from F_0_ single crosses (no more than 8 from each were randomly picked, except CG4221 mutagenesis, in which case, all F_1_ flies (4–13) from each F_0_ were picked). For the cases of *chameau* and *CG5961* mutagenesis, the ratios were calculated based on PCR-combined SmaI and HindIII digestion assays. For the efficiency comparison of TALEN- and CRISPR/Cas9-mediated HR at the *yellow* locus, F_1_ flies were randomly picked up for PCR without consideration of the *yellow* phenotype and the mutation yielders in both F_0_ and F_1_ were indicated by expected PCR bands as shown in Fig. 2B. For the statistics of the *white* platform for *yellow*, F_1_ flies with *yellow* and red eye phenotypes were scored as positive events. “Ends-in” and “ends-out” assessment was determined particularly in the case of *CG4221* mutagenesis. “Ends-in” events of *CG4221* mutagenesis were identified by the presence of a positive PCR product using the pair of primers, one located in the T7 promoter of the pBSK vector and the other in the first coding exon of *CG4221*. A list of primers used in this study is provided in supplementary material Table S4.

### Flp and Cre recombinase-mediated removal of the unwanted DNAs

To remove the 5′ part of the *white* gene in between the two *FRT* sites of the *white* platform-yielded *yellow* locus as indicated in Fig. 4, flies that carry *hs-Flp* (BL6938) on the second chromosome were crossed to the red-eyed *yellow* flies. Heat shock was induced at 37°C for 30 minutes. Images of the mosaic eyes were taken using the Leica stereo microscope. To remove the unwanted elements in *CG4221* “ends-in” events as indicated in Fig. 5A, flies carrying a *Cre* transgene on the X chromosome (BL851) were crossed to the “ends-in” mutated *CG4221* flies. The removal of the unwanted elements in the offspring of such crosses was detected by standard PCR (Fig. 5D).

### Immunofluorescence and Western blot

The protocols used for the immunostaining of GFP to detect Chameau-eGFP fusion protein, and for the detection of Myc-tagged CG4221 protein by Western blot, are as previously described (Du et al., 2010; Dui et al., 2013). The anti-GFP antibody was from Life Technologies (catalog no. A11122, 1:1000 dilution), anti-Myc was from Cell Signaling Technology (catalog no. 2276, 1:500 dilution), and the anti-tubulin antibody was from CoWin Bioscience (catalog no. CW0098, 1:5000 dilution).

## Supplementary Material

Supplementary Material
