# Supplementary material for: Various applications of TALEN- and CRISPR/Cas9-mediated homologous recombination to modify the Drosophila genome
Source: Biol Open. 2014 Mar 21;3(4):271–80. doi: 10.1242/bio.20147682 (PMC3988796; doi:10.1242/bio.20147682)
Supplement: Supplementary Material [file supp_3_4_271__index.html]

Various applications of TALEN- and CRISPR/Cas9-mediated homologous recombination to modify the Drosophila genome — Supplementary Material 

# Various applications of TALEN- and CRISPR/Cas9-mediated homologous recombination to modify the *Drosophila* genome

## bio.20147682 Supplementary Material

**Files in this Data Supplement:**

- Supplementary Material - Zhongsheng Yu et al. doi: 10.1242/bio.20147682
