# Supplementary material for: Various applications of TALEN- and CRISPR/Cas9-mediated homologous recombination to modify the Drosophila genome
Source: Biol Open. 2014 Mar 21;3(4):271–80. doi: 10.1242/bio.20147682 (PMC3988796; doi:10.1242/bio.20147682)
Supplement: Supplementary Material [file supp_bio.20147682_bio.20147682-s1.pdf]

Supplementary Material

Zhongsheng Yu et al. doi: 10.1242/bio.20147682

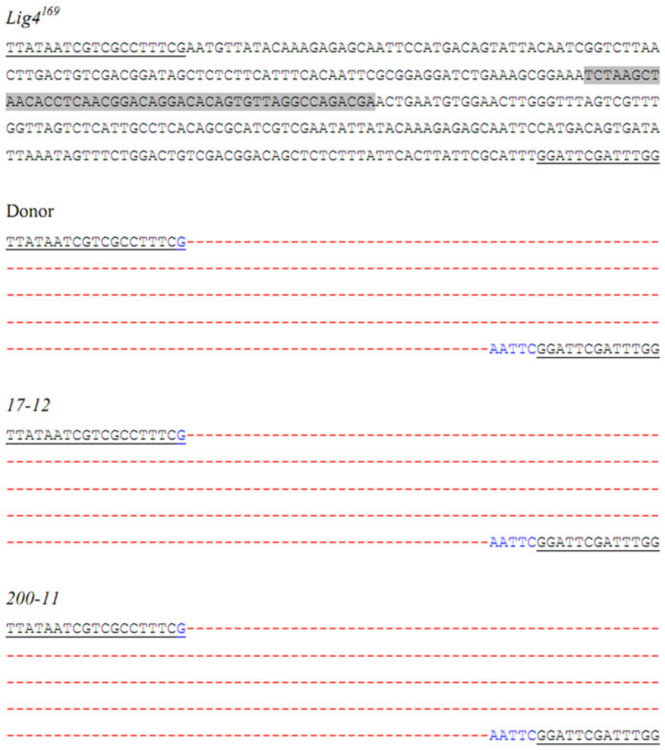

Fig. S1. Sequence analyses of *miR-281* deletion. Underlined sequences indicate the homologous parts. Sequence with grey shading indicates the pair of TALEN binding sequences and the spacer between them. The dashed line in red indicates the deleted sequence. EcoRI site: GAATTC.

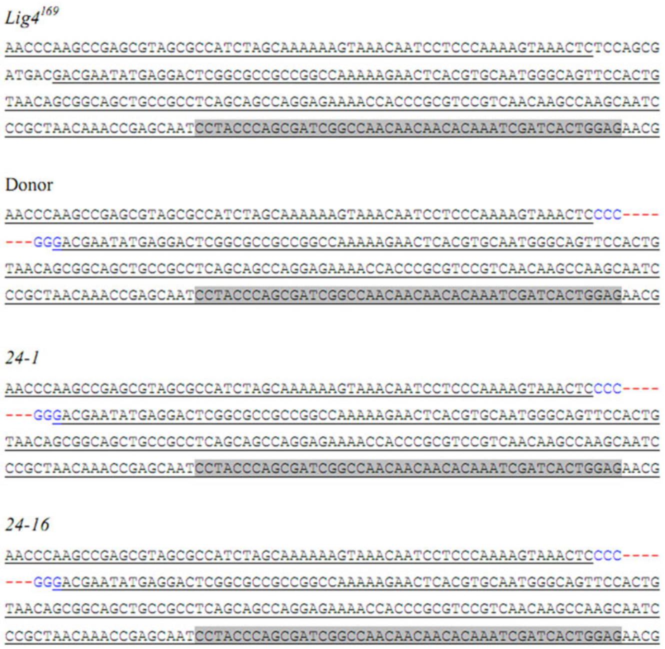

Fig. S2. Sequence analyses of *chameau* SmaI replacement. Underlined sequences indicate the homologous parts. Sequence with grey shading indicates the pair of TALEN binding sequences and the spacer between them. The dashed line in red indicates the deleted sequence. SmaI site: CCCGGG.

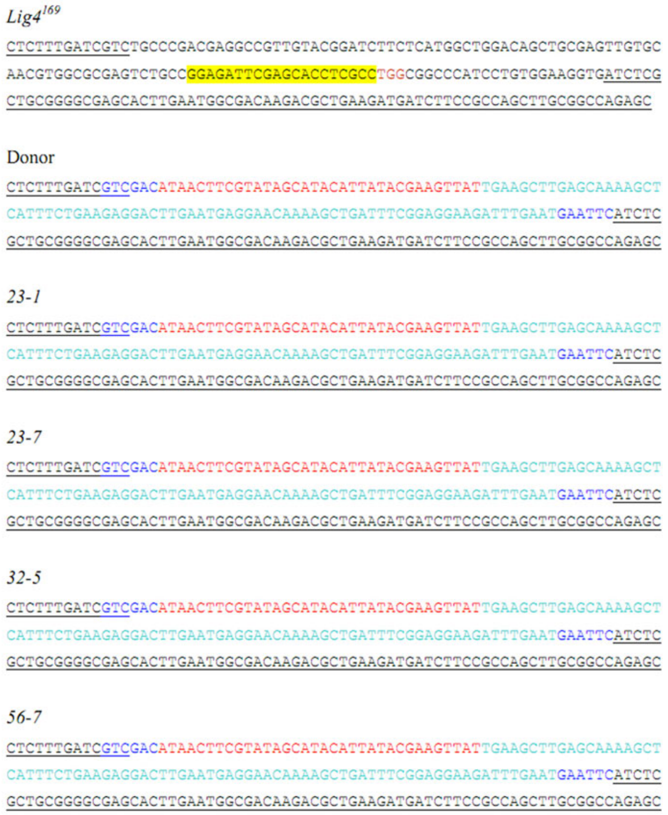

Fig. S3. Sequence analyses of *CG4221 loxP* replacement. Underlined sequences indicate the homologous parts. gRNA binding sequence and the PAM: GGAGATTCGAGCACCTCGCCTGG. *loxP* sequence: ATAACTTCGTATAGCATACATTATACGAAGTTAT. Linker sequence: TGAAGCTTGAGCAAAAGCTCATTTCTGAAGAGGACTTGAATGAGGA-ACAAAAGCTGATTTCGGAGGAAGATTTGAAT. SalI site: GTCGAC. EcoRI site: GAATTC.

Biology Open (2014) 0, 1–10 doi:10.1242/bio.20147682

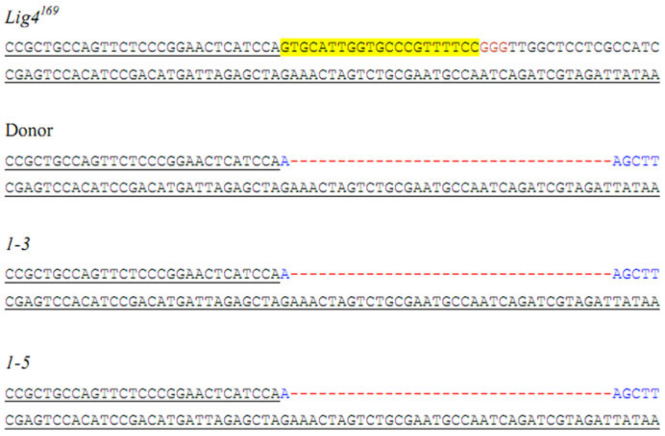

Fig. S4. Sequence analyses of *CG5961* HindIII replacement. Underlined sequences indicate the homologous parts. gRNA binding sequence and the PAM: GTGCATTGGTGCCCGTTTTCCGGG. The dashed line in red indicates the deleted sequence. HindIII site: AAGCTT.

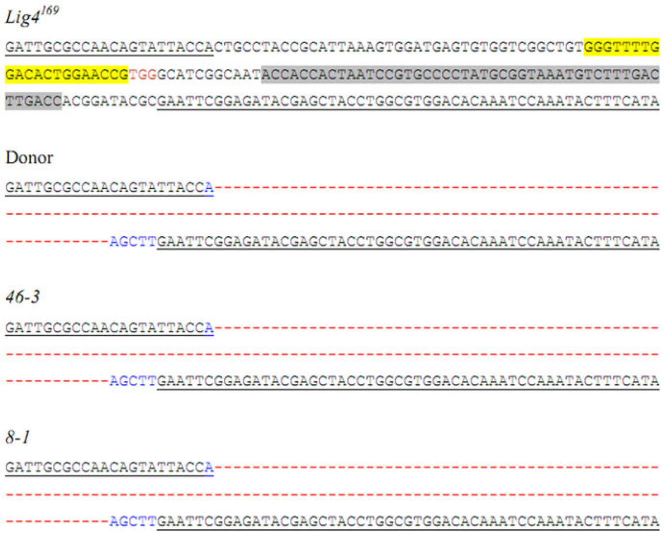

Fig. S5. Sequence analyses of the *yellow* deletion resulted from HDRs (TALEN and CRISPR/Cas9). Underlined sequences indicate the homologous parts. Sequence with grey shading indicates the pair of TALEN binding sequences and the spacer between them. gRNA binding sequence and PAM: GGGTTTTGGACACTGGAACCGTGG. The dashed line in red color indicates the deleted sequence. HindIII site: AAGCTT.

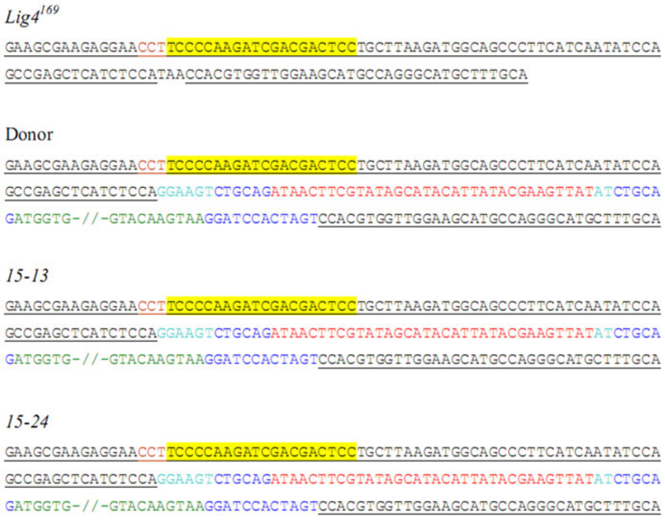

Fig. S6. Sequence analyses of *eGFP* knock-in at the *chameau* C-terminus. Underlined sequences indicate the homologous parts. gRNA binding sequence and PAM: CCTTCCCCAAGATCGACGACTCC. *loxP* sequence: ATAACTTCGTATAGCATACATTATACGAAGTTAT. *eGFP* sequence: ATGGTG-//-GTACAAGTAA. Linker sequence: GGAAGT, AT. PstI site: CTGCAG. BamHI site: GGATCC. SpeI site: ACTAGT.

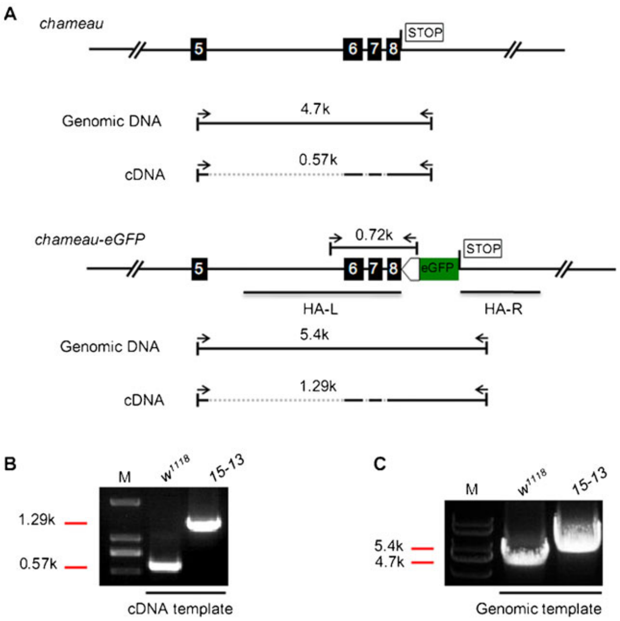

Fig. S7. Genomic and transcriptional identification of the *chameau-eGFP* fusion gene. (A) Schemes of the *chameau-eGFP* genomic and transcriptional structures. The arrows indicate the primers that were used for PCR. The genomic amplification is expected to get a 4.7 kb PCR product in w^{1118} flies, whereas a 5.4 kb PCR product in *chameau-eGFP* flies. The cDNAs amplification is expected to result in a 0.57 kb PCR product in w^{1118} flies, while a 1.29 kb PCR product in *chameau-eGFP* flies. (B,C) PCR results amplified from genomic DNAs and cDNAs. Standard genomic extraction method was employed to get the genomic DNAs from w^{1118} and *chameau-eGFP* flies. Standard RT-PCR was employed to get the cDNAs from w^{1118} and *chameau-eGFP* flies. Line *15-13* in panels B and C was used as a representative example. The legends to the elements/labels are the same as that in Fig. 3.

Biology Open (2014) 0, 1–10 doi:10.1242/bio.20147682

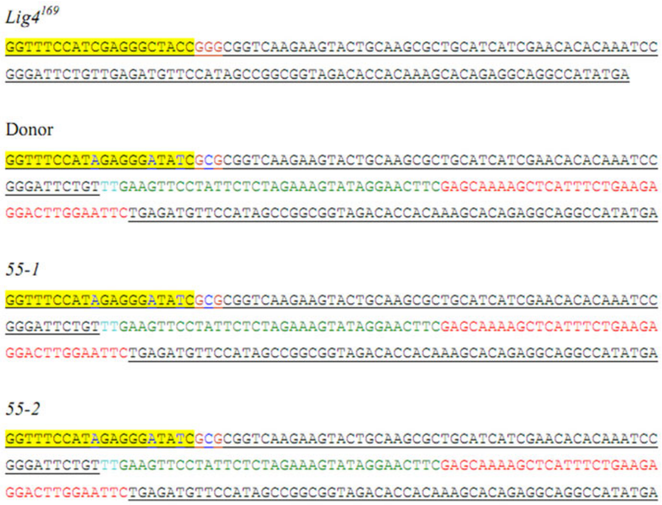

Fig. S8. Sequence analyses of *Myc* knock-in at the *CG4221* C-terminus. Underlined sequences indicate the homologous parts. gRNA binding sequence and PAM: GGTTTCCATCGAGGGCTACCGGG (the modified nucleotides are marked in blue color). FRT sequence: GAAGTTCCTATTCTCTAGAAAGTATAGGAACTTC. *Myc*-tag sequence: GAGCAAAAGCTCATTTCTGAAGAGGACTTG. Linker sequence: TT.

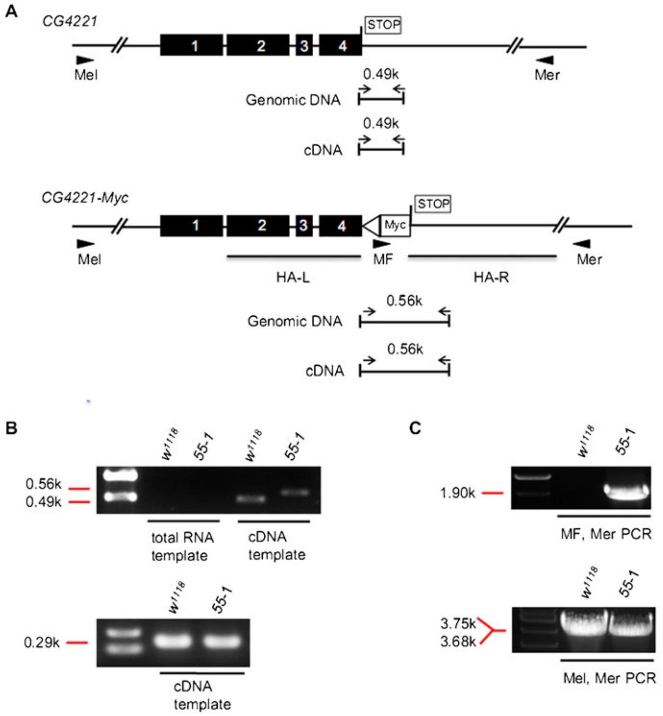

Fig. S9. Transcriptional and genomic identification of the *CG4221-Myc* fusion gene. (A) Schematic illustration of the organization of the *CG4221-Myc* fusion gene. The arrows indicate the primers used for PCRs in panel B. The arrowheads indicate the primers used for PCRs in panel C. (B) Upper panel, PCR results from cDNAs showed 0.56 kb product in *CG4221-Myc* flies and 0.49 kb product in w^{1118} flies. Standard RT-PCR was employed to get the cDNAs from w^{1118} and *CG4221-Myc* flies. Total RNA template lanes show negative result indicating no genomic DNA contamination in the RNA preparation. The amplification of *Rp49* (0.29 kb) was used as internal cDNA control (lower panel). (C) Upper panel, PCR from the genomic DNAs resulted in a 1.90 kb product in *CG4221-Myc* flies, while no PCR product in w^{1118} flies. External primer pairs, Mel and Mer, were used for genomic DNA quality control (lower panel); 3.68 kb and 3.75 kb PCR products were amplified from w^{1118} and *CG4221-Myc* flies, respectively. Line *55-1* in Fig. 3D was used as a representative example. The legends to the elements/labels are the same as that in Fig. 3.

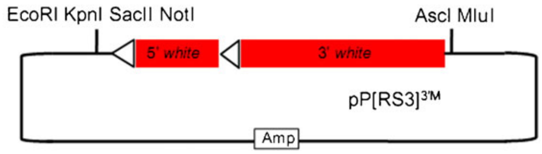

Fig. S10. The physical map of pP[RS]$^{3'M}$. The detailed construction information of pP[RS]$^{3'M}$ is shown in Materials and Methods. The left MCS (multiple cloning sites) that can be used to insert HA-L (left homologous arm) includes EcoRI, KpnI, SacII, NotI. The right MCS that can be used to insert HA-R (right homologous arm) includes AscI and MluI. Two triangles with the same orientation located in the 5′ regulatory region and the intron region of *white* gene indicate the FRT elements. Two red-filled boxes represent the 5′ *white* and 3′ *white* genomic regions.

Biology Open

Biology Open (2014) 0, 1–10 doi:10.1242/bio.20147682

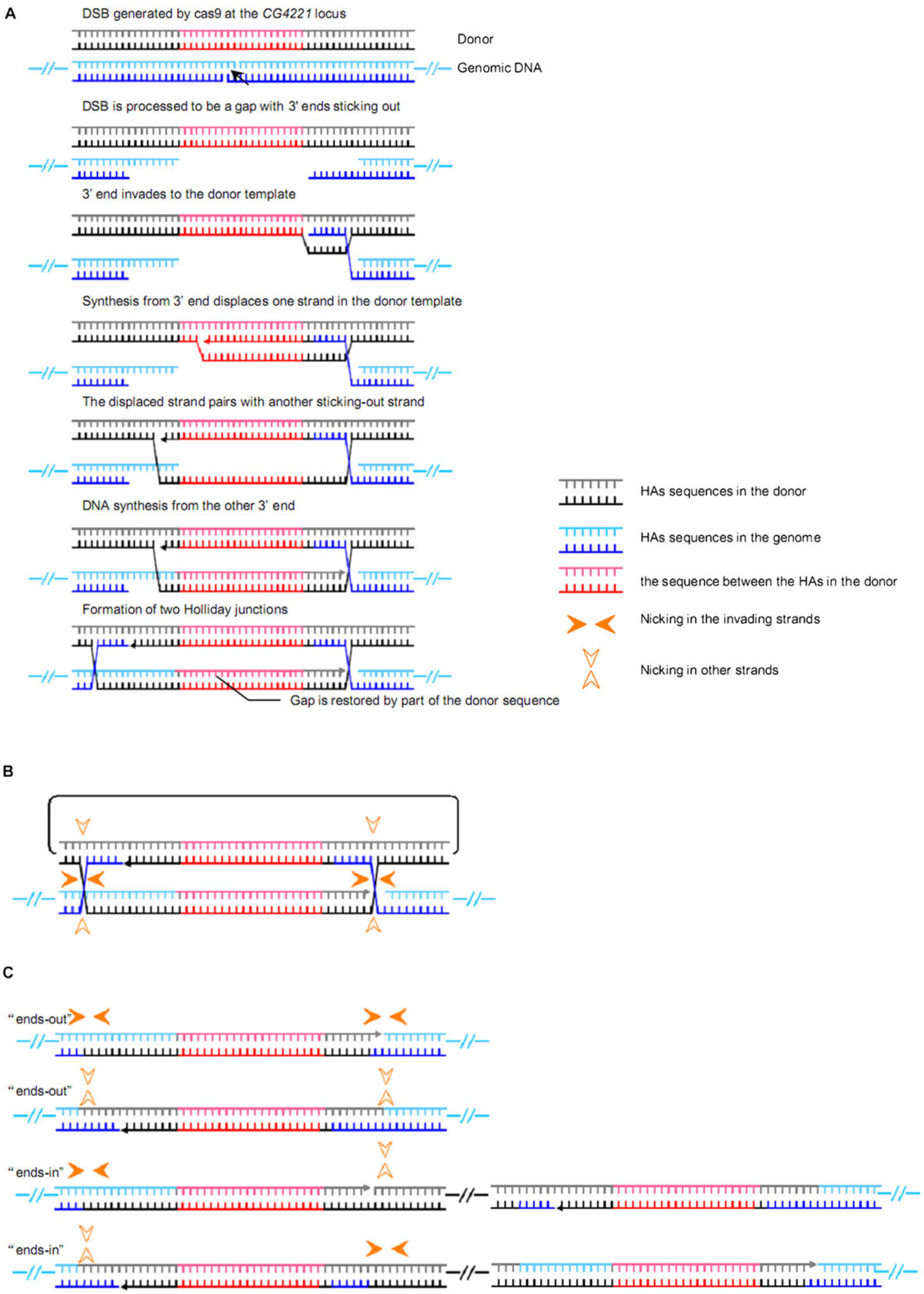

Fig. S11. Schematic illustration of *CG4221* "ends-in" and "ends out" outcomes during the CRISPR/Cas9-mediated HDR. (A) Recombination is initiated by a double-strand break and the existence of donor template. CRISPR/Cas9 cleaves the target sequence in *CG4221* locus, causing a double strand break. Using the homologous sequences in donor template, the two invading single stranded DNAs initiate DNA synthesis to repair the break, generating a second *loxP* sequence that is embedded in the red lines. (B) Two ways of resolution of the cross-overs. The intermediate repairing DNA with double Holliday junctions in the case of *CG4221*^{loxP} has to be resolved. At each cross-over, separation of the strands could occur in two ways: one is nicking at the invading strands, the other is nicking at the complementary strands. (C) Resolution of the repaired DNA generates "ends-in" or "ends-out" events in the case of *CG4221*^{loxP}. Four combinations to resolve the two cross-overs lead to two kinds of outcomes, with one copy of the *loxP* sequence left in the "ends-out" event, and two copies of the *loxP* and other sequences from the donor left in the "ends-in" event.

Biology Open

Biology Open (2014) 0, 1–10 doi:10.1242/bio.20147682

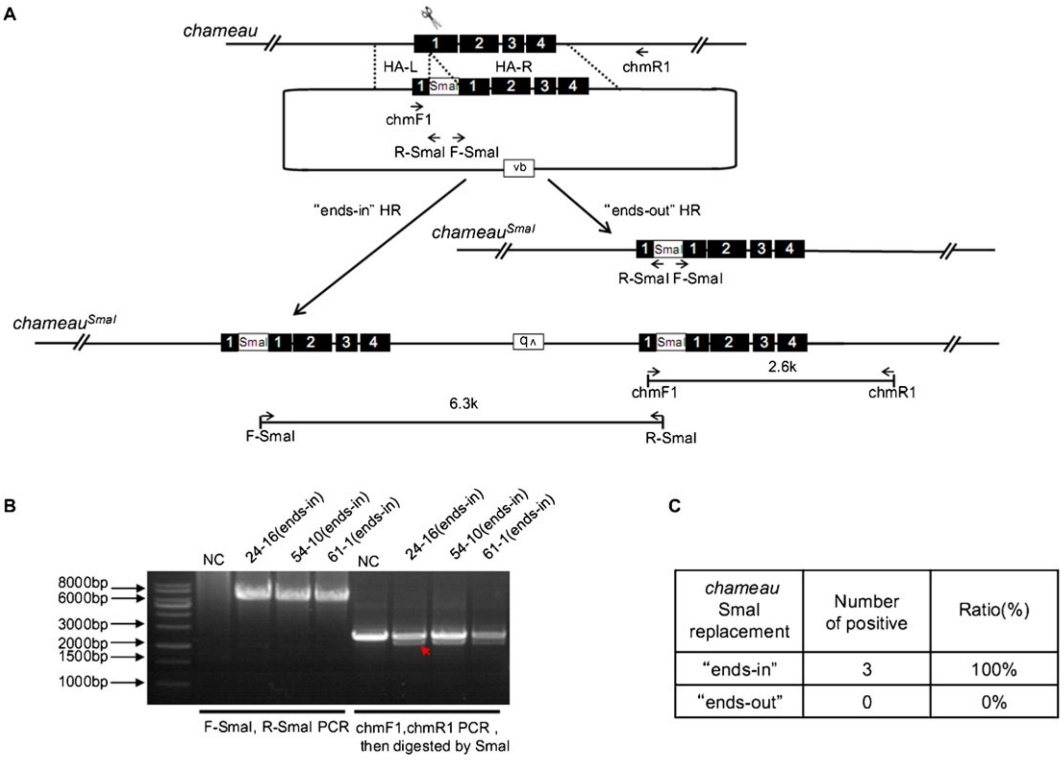

Fig. S12. Identification of the "ends-in" and "ends-out" events in *chameau* SmaI replacement. (A) "ends-in" and "ends-out" events were generated via the HDR pathway in the process of *chameau* SmaI replacement, leading to either two copies of the SmaI sites and additional sequences or one copy of the clean SmaI replacement in the fly genome. (B) PCR assays to detect the "ends-in" or "ends-out" event. The genomic DNAs of heterozyous lines *24-16*, *15-10* and *61-1* originated from independent F_0 were used. The F-SmaI and R-SmaI primers detect a corresponding 6.3 kb "ends-in" band. The chmF1 and chmR1 primers detect a 2.6 kb band containing the SmaI site, validated by the SmaI digestion. The arrows indicate the bigger bands (2.3 kb) after SmaI digestion, while the shorter/smaller bands are not shown here on the same gel. (C) "ends-in" and "ends-out" radio of the *chameau* SmaI replacement based on the PCR detection described in panel B. The legends to the elements/labels are the same as that in Figs 1 and 5.

Table S1. *Drosophila* loci with targeting sites (underlined) for TALEN-HR

Applications	TALE binding (underlined) and spacer DNA sequence
miR-281 deletion	5′ TCTAAGCTAACACCTCAACGGACAGGACACAGTGTTAGGCCAGACGA 3′<br>3′ AGATTCGATTGTGGAGTTGCCTGTCCTGTGTCACAATCCGGTCTGCT 5′
chameau SmaI replacement	5′ TCCTACCCAGCGATCGGCCAACAACAACACAAATCGATCACTGGAGA 3′<br>3′ AGGATGGGTCGCTAGCCGGTTGTTGTTGTGTTTAGCTAGTGACCTCT 5′
yellow deletion	5′ TACCACCACTAATCCGTGCCCCTATGCGGTAAATGTCTTTTGACTTGACCA 3′<br>3′ ATGGTGGTGATTAGGCACGGGGATACGCCATTTACAGAAAACTGAACTGGT 5′
white knock-in at the *yellow* locus	the same as *yellow* deletion

Table S2. *Drosophila* loci with targeting sites chosen for Cas9-HR and oligonucleotides used to generate the corresponding gRNAs

Applications	Target sequence (5′ to 3′) (PAM is underlined)	Forward oligonucleotide (5′ to 3′)	Reverse oligonucleotide (5′ to 3′)
CG4221 loxP replacement	GGAGATTCGAGCACCTCGCCTGG	TAATACGACTCACTATAGGAGATTCGAGCA-CCTCGCCGTTTTAGAGCTAGAAATAGC	AAAAAAAGCACCGAC-TCGGTGCCAC
CG5961 HindIII replacement	GGAAAACGGGCACCAATGCACTGG	TAATACGACTCACTATAGGAAAACGGGCAC-CAATGCACGTTTTAGAGCTAGAAATAGC	the same as above
yellow deletion	GGGTTTTGGACACTGGAACCGTGG	TAATACGACTCACTATAGGGTTTTGGACAC-TGGAACCGGTTTTAGAGCTAGAAATAGC	the same as above
Chameau C-terminal eGFP tagging	GGAGTCGTCGATCTTGGGGAAGG	TAATACGACTCACTATAGGAGTCGTCGATC-TTGGGGAGTTTTAGAGCTAGAAATAGC	the same as above
CG4221 C-terminal Myc tagging	GGTTTCCATCGAGGGCTACCGGG	TAATACGACTCACTATAGGGTTTCCATCGA-GGGCTACCGTTTTAGAGCTAGAAATAGC	the same as above

Biology Open (2014) 0, 1–10 doi:10.1242/bio.20147682

Table S3. Primers used for donor constructions.

Applications	Primers for left homologous arm amplification (5′ to 3′)	Primers for right homologous arm amplification (5′ to 3′)	Primers used for amplifying additional elements (5′ to 3′)
miR-281 deletion	F: CAGACAggatccCACCAA-GCTAGCGAAC<br>R: TGTATAgaattcATTCGA-AAGGCGACGATT	F: TATTCgaattcGGAT-TCGATTTGGTTCG<br>R: AGTTActcgagGA-ACTGAATGTGGAACT	–
chameau SmaI replacement	F: AGGaagcttCTGTCCTG-TACCACTGACGT<br>R: AAcccgggAGTTTACTT-TTGGGAGGATTG	F: ATcccgggACGAA-TATGAGGACTCGGC<br>R: ATtctagaGAGCAG-GATCAGATTTTCCGC	–
CG4221 loxP replacement	F: GTCggtaccAGCTTGG-CACACATATTGC<br>R: TATgtcgacACGATCA-AAGAGCGGACCGC	F: TTTgaattcATCTC-GCTGCGGGGCGAGCA<br>R: GGGactagtTGCCT-GCCACTTCAT CAACT	loxP-F: tcgacATAACTTCGTATAGCATACATTATACGAAGTTATTGa<br>loxP-R: agcttCAATAACTTCGTATAATGTATGCTATACGAAGTTATg<br>linker-F: agcttGAGCAAAAGCTCATTTCTGAAGAGGACTTGAAT-GAGGAACAAAAGCTGATTTCGGAGGAAGATTTGAATG<br>linker-R: aattcATTCAAATCTTCCTCCGAAATCAGCTTTTGTTC-CTCATTCAAGTCCTCTTCAGAAATGAGCTTTTGCTCa
CG5961 HidIII replacement	F: GATCggtaccCTAAG-CCAATGGTGCATCAGC<br>R: GCGCaagcttTGGA-TGAGTTCCGGGAGAACT	F: GCGCaagcttCGAG-TCCACATCCGACATGAT<br>R: ATATccgcggGAAT-TCCAGTTGCCGTATCGG	–
yellow deletion	F: ATGCggtaccAGTCC-GAAACACCCAAACTTC<br>R: GCGCaagcttGGTAATA-CTGTTGGCGCAATC	F: GCGCaagcttGAAT-TCGGAGATACGAGCTAC<br>R: GCGCtctagaACCT-TGATGCTGATGATGCC	–
Chameau C-terminal eGFP tagging	F: AACggtaccTGTTAGA-GCGGTACGGCCATACACC<br>R: GGCctgcagACTTCCTG-GAGATGAGCTCGGCTG-GATATTGA	F: GGactagtCCACGT-GGTTGGAAGCATGCCA<br>R: AAgcggccgcCTGT-TGGCGATCAAATGCTG AATGTCGG	eGFP-F: TActgcagATGGTGAGCAAGGGCGAGGA<br>eGFP-R: CGggatccTTACTTGTACAGCTCGTCCA<br>loxP-F: gATAACTTCGTATAGCATACATTATACGAAGTTATATctgca<br>loxP-R: gATATAACTTCGTATAATGTATGCTATACGAAGTTATctgca
CG4221 C-terminal Myc tagging	F: CTTgtcgacGAGCGACTG-CCAGGTGGGT<br>R: CGCgatatcCCTCTATG-GAAACCGGACAGTCCTGGA	F: CACgaattcTGAGA-TGTTCCATAGCCGGC<br>R: TTAggatccTCACCT-GTGCGCAGGTTGGA	F: AGGgatatcGCGCGGTCAAGAAGTACTGCAAGCGCTGCATCAT-CGAACACACAAATCCGGGATTCTGTTTGAAGTTCCTATTCTCTAG<br>R: CATgaattcCAAGTCCTCTTCAGAAATGAGCTTTTGCTCGAAGT-TCCTATACTTTCTAGAGAATAGGAACTTCAAACAGAATCCCGGA
white knock-in at the *yellow* locus	F: CTCATAAATCCGGTT-ggtaccTGC<br>R: TATAAGAATgcggccgc-GATTAGTGGTGGTAT-TGCCGATGC	F: ATATggcgcgccGTCTTTG-ACTTGACCACGGATAC<br>R: ATGCacgcgtTGAAGT-CTGGCTCCGATCAAAAG	–

Forward (F) and reverse (R) primers. Restriction sites are in lower case.

Biology Open (2014) 0, 1–10 doi:10.1242/bio.20147682

Table S4. Primers used for molecular identifications

Applications	Primers (5′ to 3′)<br>forward (F) and reverse (R)
miR-281 deletion	F: GAAAGAGGCAGACGTTGTTTG<br>R: CTGGCATATTTCGCACGTAG
chameau SmaI replacement	F: TCTGCGCTACAAAAATGGCC<br>R: GGTTTGTTAGCGGGATTGCT
CG4221 loxP replacement	loxPf: TCGACATAACTTCGTATAGCATACATTATACGAAGTTATTGA<br>R: TATGTCGACACGATCAAAGAGCGGACCGC
CG5961 HindIII replacement	F: TGCTCGTAGAACCTGAACTC<br>R: CCCAAAATCAGCAATACGCG
yellow deletion	F: GGCAGTTACCATTGCTTATG<br>R: GATCGAATGGGCGAAAGGGACATA
Chameau C-terminal eGFP tagging	F: ACGAACACACTTAAGCACCC<br>R: GATATAACTTCGTATAATGTATGCTATACGAAGTTATCTGCA
CG4221 C-terminal Myc tagging	F: ATCTGAACGCTCGAGGATGT<br>R: CCCAAAAGTCCGTGTATGGCT
white knock-in at the *yellow* locus	HA-L-F: GGCAATCGGTTATTCGTCAC<br>HA-L-R: CGTTAACGTTCGAGGTCGACTCTAG<br>HA-R-F: CTCAAATGGTCCGAGTGGT<br>HA-R-R: ACGACTACAGGCTCACAATC
"ends-in" and "ends-out" events in *CG4221 loxP* replacement	T7f: GTAATACGACTCACTATAGGGC<br>e1r: TAGTGGTCGCGTACCGCATTG<br>er: TGCTACGCTAGTTCTGTCCTG<br>loxPf: TCGACATAACTTCGTATAGCATACATTATACGAAGTTATTGA<br>Rp49-G-F: ACGTTGTGCACCAGGAACTT<br>Rp49-G-R: TACAGGCCCAAGATCGTGAA
genomic and transcriptional identification of the *chameau-eGFP*	F: AGGGTTACGGAAGATTACTCATCG<br>R: CCGCTAGGATAAATGCAAAGG
genomic and transcriptional confirmation of the *CG4221-Myc*	F: ATCTGAACGCTCGAGGATGT<br>R: CCCAAAAGTCCGTGTATGGCT<br>Rp49F: TATGCTAAGCTGTCGCACAA<br>Rp49R: ACCAGGAACTTCTTGAATCC
genomic and transcriptional confirmation of the *CG4221-Myc*	MF: ATCTGAACGCTCGAGGATGT<br>MeI: TCAGTGACATAGACCGTAACG<br>Mer: ATAGATAGCATGACTGTGTCC
"ends-in" and "ends-out" events in *chameau* SmaI replacement	chmF1: TCTGCGCTACAAAAATGGCC<br>chmR1: TCCGTCTCCAGACATAAAGG<br>F-SmaI: ATCCCGGGACGAATATGAGGACTCGGC<br>R-SmaI: AACCCGGGAGTTTACTTTTGGGAGGATTG

Biology Open
